# Ushering in safe, effective, secure, and ethical medicine in the digital era

**DOI:** 10.1038/s41746-021-00424-5

**Published:** 2021-03-25

**Authors:** William J. Gordon, Andrea R. Coravos, Ariel D. Stern

**Affiliations:** 1 Brigham and Women’s Hospital, Boston, MA USA; 2 Harvard Medical School, Boston, MA USA; 3 Mass General Brigham, Boston, MA USA; 4 Elektra Labs, Inc., San Francisco, CA USA; 5 Harvard-MIT Center for Regulatory Science, Boston, MA USA; 6 Harvard Business School, Boston, MA USA; 7 Health Innovation Hub, German Federal Ministry of Health, Berlin, Germany

**Keywords:** Diagnosis, Therapeutics, Biomarkers

## Abstract

From clinical trials to care delivery, advanced, digitally enabled technologies and analytics offer new approaches to how we think about medicine, health, and biology. The Covid-19 pandemic has accelerated this conversation, and forced a roadmap, once measured in years or decades, to unfold over days, weeks, and months. Yet the scaffolding for this roadmap had already emerged prior to the Covid-19 pandemic. In this perspective, we highlight a special collection of papers on “digital medicine,” which emerged from a symposium held in Boston in 2019 and were published in 2020 and 2021. The symposium was hosted by Harvard Business School and the Harvard MIT Center for Regulatory Science, and included a range of speakers and attendees from industry, government, and academics. We describe their ongoing relevance as we contemplate our early 2021 pandemic reality and the near future of digitally empowered health care.

## What is digital medicine?

Digital medicine describes an evidence-based field, concerned with the use of technologies as tools for measurement and intervention in the service of human health^1^. Digital medicine products are driven by high-quality hardware and software products that support health research and the practice of medicine broadly, including treatment, recovery, disease prevention, and health promotion for individuals and across populations^2^. As described below, we are witnessing the emergence of robust, evidence-based, technologies that have come to maturity and are solving intractable clinical problems. Digital medicine tools can (but need not) be integrated into the formal health care delivery system, a process that provides unique challenges and vital opportunities, as documented in this collection of articles.

## Evaluating digital medicine

One of the early themes of the symposium was understanding the current state of evidence vis-à-vis digital medicine tools. While the potential for such tools to improve care delivery, research, and operations was clear, a critical first question is “how do we evaluate this tool?” In “Modernizing and designing evaluation frameworks for connected sensor technologies in medicine,” Coravos et al.^3^ look at connected sensors in particular, and highlight five dimensions of benefit–risk—(1) validation, (2) security practices, (3) data rights and governance, (4) utility and usability, and (5) economic feasibility. The authors provide a comprehensive review of many of the current evaluation frameworks for connected technologies and close by building on some of these current themes to propose a novel evaluation framework that builds upon the five dimensions described.

In “Verification, analytical validation, and clinical validation (V3): the foundation of determining fit-for-purpose for Biometric Monitoring Technologies (BioMeTs),” Goldsack et al.^4^ focus on the first step of the above framework, validation. The authors focus on Biometric Monitoring Technologies, or BioMeTs, which are defined as digital medicine products that process the data generated or captured by mobile sensors, using algorithms to generate measures of behavioral and/or physiological function. The interdisciplinary, international group of authors outlines a three-component evaluation framework for BioMeTs, notably including verification, analytical validation, and clinical validation (the “V3 Framework”).

Finally, in “Investigating sources of inaccuracy in wearable optical heart rate sensors,” Bent et al.^5^ look closely at a specific type of digital tool—optical sensors for determining heart rate, and compare six different wearable devices across 53 study participants. While authors did not detect differences in accuracy across skin tones, they did find inconsistencies between devices, and that error was particularly noted during activity. Future work in this area—quantifying the diagnostic accuracy of digital medicine tools—will be essential for understanding their utility and diagnostic test characteristics.

Understanding the dimensions of benefit–risk, practical challenges, terminology, and frameworks outlined in these articles will only continue to grow in importance as connected sensors are expected to play a growing role in both health care delivery and clinical research^6,7^.

## Current usage of digital medicine tools

A second theme that emerged from the symposium was understanding real-world use cases and patterns in usage for digital medicine tools. In “Quantifying the use of connected digital products in clinical research,” Marra et al.^8^ performed a systematic search of the ClinicalTrials.gov database to identify and quantify the use of connected devices in clinical research over time. The authors found a clear increase in their use over the past two decades, with their findings pointing to a roughly 34% cumulative annual growth rate in the incorporation of connected digital products into registered clinical trials. Notably, this analysis was performed on data that predate the COVID-19 pandemic and therefore do not yet account for any additional adoption that may have been catalyzed by it^9^. Moreover, the authors find that these products have been used by a diverse group of sponsors and across all phases of clinical trials, suggesting the broad applicability of this technology to clinical research.

## Security and data rights

A third theme that emerged was around safeguarding patient data as a safety issue, with two main perspectives: (a) theft as a “data security” issue, and (b) misuse as a “data rights” issue. In “Building resilient medical technology supply chains with a software bill of materials,” Carmody et al.^10^ outline how an exploited vulnerability in a single software component of health care technology can affect patient care and how to manage that risk by publishing a digital medicine product’s software bill of materials (SBOM). SBOMs, which have garnered FDA support in recent guidance documentation^11^, have the potential to benefit all supply chain stakeholders of medical technologies and the increased transparency can unlock and enable trustworthy, resilient, and safer health care technologies for all.

In “Privacy protections to encourage use of health-relevant digital data in a learning health system,” McGraw and Mandl^12^ highlight gaps in US health privacy laws that do not cover data collected by many consumer digital technologies and which have not been updated to address concerns about large technology companies entering the health care industry. The authors provide pragmatic recommendations for protecting individuals by establishing rules for health-relevant data that go beyond relying only on consent.

## Last mile and implementation

Finally, the symposium participants discussed how real-world factors such as patient attrition could impact the strength and generalizability of digital medicine studies, as well as the health care provider’s perspective on the use and dissemination of digital tools.

In “Indicators of retention in remote digital health studies: a cross-study evaluation of 100,000 participants,” Pratap et al.^13^ highlight the operational issue of patient retention as particularly salient. The article takes a solution-oriented stance, describing correlates of retention (and thus as a corollary, attrition) in a set of eight studies that were analyzed in detail. While much more work is needed to understand the factors that shape patient retention in clinical trials in digital medicine, this piece outlines a set of factors such as compensation, illness, age, and clinician involvement that predict continuation and are therefore likely to be helpful in designing more effective recruitment and retention strategies in the future. As in the other articles in this collection, this study’s findings are of growing relevance in a rapidly digitizing research ecosystem.

In “Beyond validation: getting health apps into clinical practice,” Gordon et al.^14^ move past validation, and take on the logistics and practicalities of disseminating “apps” into clinical practice, by proposing a framework for prescribing apps, akin to how clinicians prescribe medications or other devices today. This includes the creation of digital formularies, workflow and EHR integration, payment models, and various forms of educational support, like training and awareness. Importantly, equity concerns abound, and heavier reliance on “apps” for care delivery may exacerbate an extant digital divide.

As a response to Gordon et al., Cohen and Martin’s^15^ editorial “Innovation without integration” notes that heavily integrated apps are only one component of the larger ecosystem, and many technologies, like prevention-focused apps, may be able to avoid the complexity of EHR integration entirely while still providing immense value. Indeed, the authors argue, that while full integration with electronic health records may take years, those tools that do not demand such integration can be deployed relatively quickly.

As highlighted by these pieces, bringing digital medicine to the patient is complex, with numerous dependencies, including technical work, payment incentive alignments, patient engagement and retention, and education. Digital medicine will likely be most successful and create the most value^16^ when it is woven into the care continuum, alongside traditional interventions like medications and medical devices.

## Discussion

The field of digital medicine is moving quickly, with new users, use cases, technical capabilities, data sources, and analytical capabilities, rapidly emerging and evolving. In some cases, the studies discussed here represent a first “stake in the ground” in bringing data to bear on questions raised by digital medicine. Understanding the phenomena highlighted in this collection will be of great importance for researchers, clinicians, and policy makers worldwide. For example, understanding factors associated with study retention in other settings will help with research design, while understanding the use of use cases for connected digital tools in clinical research will be of great value during ongoing Covid-19 public health emergency and beyond. Furthermore, much work is needed to bolster cybersecurity and data rights considerations, warranting significant investment, discussion, and research going forward.

Collaboration with regulators will be vital to ensuring a robust scaffolding for the development of safe and effective digital medicine products. Fortunately, since the time of the symposium, regulatory innovation has continued apace, with encouraging developments on both sides of the Atlantic. In the United States, the FDA recently announced a Digital Health Center of Excellence^17^, and in Germany, the Digital Healthcare Act (Digitale-Versorgung-Gesetz) rapidly expands reimbursement opportunities for certain digital health applications^18^, as just two examples.

Digital medicine continues to rapidly evolve. A rigorous and consistent foundation for understanding digital medicine is therefore critical for framing and ushering these advancements safely into practice and to meet the patient where they are at. We welcome this evolution, and the myriad ways in which digital medicine will improve our understanding, management, and treatment of human disease, and become “just plain medicine.”^19^

## References

[1] Digital Medicine Society. Defining Digital Medicine. *Digit Biomark*. **3**, 31–71 https://www.karger.com/Article/FullText/500413#ref4 (2019). 10.1159/000500413 PMC7015383 32095767

[2] Coravos A (2019). Digital medicine: a primer on measurement. Digit. Biomark..

[3] Coravos A (2020). Modernizing and designing evaluation frameworks for connected sensor technologies in medicine. npj Digit. Med..

[4] Goldsack JC (2020). Verification, analytical validation, and clinical validation (V3): the foundation of determining fit-for-purpose for Biometric Monitoring Technologies (BioMeTs). npj Digit. Med..

[5] Bent B, Goldstein BA, Kibbe WA, Dunn JP (2020). Investigating sources of inaccuracy in wearable optical heart rate sensors. npj Digit. Med..

[6] Sanders, S., Stern, A. & Gordon, W. How to make remote monitoring tech part of everyday health care. https://hbr.org/2020/07/how-to-make-remote-monitoring-tech-part-of-everyday-health-care (2020).

[7] McFarling, U. L. ‘Why would we ever go back?’: Covid-driven shift to remote cancer clinical trials will likely outlast the pandemic. STAT. https://www.statnews.com/2020/12/09/shift-to-remote-cancer-clinical-trials-will-likely-outlast-pandemic/. Accessed on 3/8/2021.

[8] Marra C, Chen JL, Coravos A, Stern AD (2020). Quantifying the use of connected digital products in clinical research. npj Digit. Med..

[9] FDA. Conduct of clinical trials of medical products during the COVID-19 public health emergency: Guidance for Industry, Investigators, and Institutional Review Boards. https://www.fda.gov/media/136238/download (2020).

[10] Carmody, S. et al. Building resilient medical technology supply chains with a software bill of materials. *npj Digit. Med*. 10.1038/s41746-021-00403-w (2021). 10.1038/s41746-021-00403-w PMC7902663 33623135

[11] FDA. Guidance for the content of premarket submissions for software contained in medical devices. https://www.fda.gov/downloads/MedicalDevices/…/ucm089593.pdf (2005).

[12] McGraw D, Mandl KD (2021). Privacy protections to encourage use of health-relevant digital data in a learning health system. npj Digit. Med..

[13] Pratap, A. et al. Indicators of retention in remote digital health studies: a cross-study evaluation of 100,000 participants. *npj Digit. Med*. **3**, 21 10.1038/s41746-020-0224-8 (2020). 10.1038/s41746-020-0224-8 PMC7026051 32128451

[14] Gordon, W. J., Landman, A., Zhang, H. & Bates, D. W. Beyond validation: getting health apps into clinical practice. *npj Digit. Med*. **3**, 14 10.1038/s41746-019-0212-z (2020). 10.1038/s41746-019-0212-z PMC6997363 32047860

[15] Cohen, A. B. & Martin, S. S. Innovation without integration. *npj Digit. Med*. **3**, 15 10.1038/s41746-020-0220-z (2020). 10.1038/s41746-020-0220-z PMC6997154 32025575

[16] Ganguli, I. et al. Machine learning and the pursuit of high-value health care. *NEJM Catal*. **1** (2020).

[17] FDA. FDA Launches the Digital Health Center of Excellence. https://www.fda.gov/news-events/press-announcements/fda-launches-digital-health-center-excellence (2020).

[18] Gerke, S., Stern, A. D. & Minssen, T. Germany’s digital health reforms in the COVID-19 era: lessons and opportunities for other countries. *npj Digit. Med*. **3**, 94 10.1038/s41746-020-0306-7 (2020). 10.1038/s41746-020-0306-7 PMC7351985 32685700

[19] Steinhubl SR, Topol EJ (2018). Digital medicine, on its way to being just plain medicine. npj Digit. Med..

